# Identification of Pns6, a putative movement protein of RRSV, as a silencing suppressor

**DOI:** 10.1186/1743-422X-7-335

**Published:** 2010-11-22

**Authors:** Jianguo Wu, Zhenguo Du, Chunzheng Wang, Lijun Cai, Meiqun Hu, Qiying Lin, Zujian Wu, Yi Li, Lianhui Xie

**Affiliations:** 1 Institute of Plant Virology, Fujian Agriculture and Forestry University , Key Laboratory of Plant Virology of Fujian Province, Fuzhou, Fujian, China; 2 Peking-Yale Joint Center for Plant Molecular Genetics and Agrobiotechnology, The National Laboratory of Protein Engineering and Plant Genetic Engineering, College of Life Sciences, Peking University, Beijing China

## Abstract

RNA silencing is a potent antiviral response in plants. As a counterdefense, most plant and some animal viruses encode RNA silencing suppressors. In this study, we showed that Pns6, a putative movement protein of *Rice ragged stunt virus *(RRSV), exhibited silencing suppressor activity in coinfiltration assays with the reporter green fluorescent protein (GFP) in transgenic *Nicotiana benthamiana *line 16c. Pns6 of RRSV suppressed local silencing induced by sense RNA but had no effect on that induced by dsRNA. Deletion of a region involved in RNA binding abolished the silencing suppressor activity of Pns6. Further, expression of Pns6 enhanced *Potato virus × *pathogenicity in *N. benthamiana*. Collectively, these results suggested that RRSV Pns6 functions as a virus suppressor of RNA silencing that targets an upstream step of the dsRNA formation in the RNA silencing pathway. This is the first silencing suppressor to be identified from the genus *Oryzavirus*.

## 

Plant infecting reoviruses are grouped into three genera, namely *Phytoreovirus, Fijivirus *and *Oryzavirus *[1]. RRSV belongs to the genus *Oryzavirus*. It infects plants in the family *Graminae *and is transmitted in a persistent manner by brown plant hoppers. The disease caused by this virus was first discovered in 1976-1977 in Indonesia and Philippines. Then the disease became prevalent in most rice-growing countries in south-eastern and far-eastern Asia, causing great yield losses to rice production [2]. RRSV virion has an icosahedral particle which consists of a polyhedral core surrounded by flat spikes about 20 nm wide and 10 nm high [3]. The RRSV genome comprises 10 double stranded RNAs with molecular weights ranging from 1.2 to 3.9 kb [4]. The complete nucleotide sequences of all genomic segments, denoted as S1-S10, have been determined. It is now known that proteins encoded by S6, S7 and S10, with *Mrs *of about 71 kD, 68KD and 32KD, respectively, are non-structural proteins, whereas proteins specified by other RNA segments have been shown to be present in or are supposed to take part in the assembly of RRSV virions [5-13]. However, our knowledge on the roles played by RRSV encoded proteins in virus-host interaction remains poor.

RNA silencing is now a general term that refers to a set of related processes in which small RNAs ranging from 21 to 30nt in length are used to direct sequence specific modulation of gene expression [14]. In plants as well as in some animals, one of the firmly established roles of RNA silencing is antiviral defense [15,16]. The antiviral RNA silencing begins with the cleavage of viral dsRNAs by members of the RNase III family enzymes called Dicer or Dicer-like (DCL) in plants, which results in the production of viral small interfering RNAs (VsiRNAs). These vsiRNAs are incorporated into an effector complex named RNA induced silencing complex (RISC) and then direct the complex to destroy viral RNAs [15,16]. VsiRNAs also provide sequence specificity for cellular RDRs to copy viral RNAs into dsRNAs, which can be a secondary source for vsiRNAs production [17,18]. As a counterdefence, many viruses have evolved to encode one or multiple proteins to suppress RNA silencing [15,19]. The molecular mechanisms by which these virus encoded silencing suppressors (VSRs) interfere with the RNA silencing machinery are poorly understood at present. VSRs from diverse viruses posses RNA binding activities. This led to the proposition that RNA might be a common target of VSRs. [20,21]. Some VSRs such as p19, HC-Pro and p21 efficiently form complexes with 21-nt ds-sRNA but fail to bind long dsRNA. These VSRs were believed to prevent the formation of functional RISCs by sequestering vsiRNAs. Some VSRs including CP of *Turnip crinkle virus *(TCV) bind dsRNAs without size selection. These VSRs may function to protect viral dsRNAs from being cleaved by plant DCLs [20,21]. Many recent studies demonstrated that VSRs could also target protein components of RNA silencing [for a review see 22]. Protein components of the RNA silencing that have been found to be targets of VSRs include DRB4, which is an auxiliary factor of the antiviral sensor DCL4; AGO1, which forms the core of RISC; and SGS3 which is a cofactor of RDR6 functioning in the amplification step of RNA silencing [22]. Regardless of all these possibilities, it is believed that VSRs play important roles in promoting viral replication as well as in viral pathogenesis [23].

The VSRs for members of the other two genera of Phytopathogenic reoviruses have been identified [24-26]. However, no VSR for *Oryzavirus *has been reported, nor can it be predicted because of the low level of sequence similarity between proteins of reoviruses across genera. Given the importance of VSRs in virus-host interaction, we conducted experiments to identify the VSR of RRSV.

It was reasonable to presume that the silencing suppressor function of RRSV was encoded by a non-structural protein. Therefore, the ORFs for the three non-structural proteins, pns6, pns7 and pns10, were individually cloned into the binary vector pPZP212(See Additional file 1 and 2 for Materials and Methods). The resultant plasmids were named 35S-S6, 35S-S7, and 35S-S10 respectively (Figure 1). Transformed agrobacterial strain carrying each of these constructs was mixed with a strain that carried 35S-GFP with a ratio of 3:1 and infiltrated into leaves from *Nicotiana benthamiana *line 16c as described previously [27]. Then the GFP fluorescence was monitored using a handheld long wavelength UV light source. Agrobacteria harboring only the GFP gene or the 2b gene of *Tomato aspermy cucumovirus *(TAV) were used as negative and positive controls, respectively [28].

**Figure 1:**
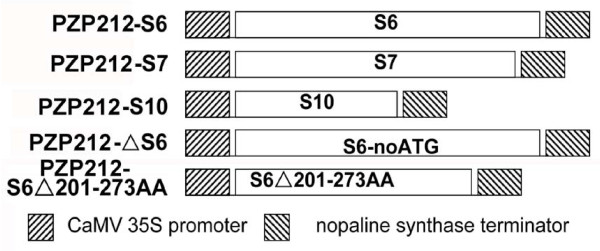
**Schematic representation of plasmids used in this study**.

All infiltrated leaf patches showed bright green fluorescence at 2 days post infiltration (dpi.) **(Data not shown)**. In leaf patches infiltrated with 35S-GFP plus 35S-S6 or plus 35S-2b, the fluorescence intensity remained strong until 7dpi (Figure 2A, C). However, in leaf patches expressing GFP or GFP plus either S7 or S10, the fluorescence intensity began to decline at 3dpi and became hardly detectable at 7dpi (Figure 2D,E). Similar patterns of fluorescence decline were observed in leaf patches infiltrated with 35S-GFP plus 35S-ΔS6 (Figure 2B). The 35S-ΔS6 carried the same S6 gene sequence as 35S-S6, but was supposed to be unable to express a functional Pns6 protein because of the deletion of the nucleotide A from the translation start codon AUG (Figure 1).

To test whether silencing suppression was responsible for the above observations, Northern blot analyses were conducted to detect steady-state levels of GFP mRNA and GFP-specific siRNAs. As shown in Figure 2G, the accumulation levels of GFP mRNA were much higher in tissues expressing 35SGFP plus 35S-TAV2b or 35S-GFP plus 35S-S6 than in tissues expressing GFP alone or in combination with any other genes at 7 dpi. This indicated that expression of S6 resulted in the stabilization of GFP mRNA and consequently higher GFP fluorescence, as that of TAV2b. In all treatments, the accumulation levels of GFP mRNA were negatively correlated with those of GFP-specific siRNAs (Figure 2G). This confirmed that Pns6 is a silencing suppressor.

**Figure 2:**
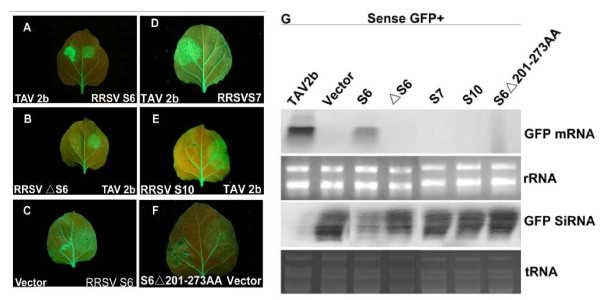
**Suppression of local GFP silencing by RRSV Pns6**. (A-F) *N. benthamiana *line 16c plants were coinfiltrated with *Agrobacterium *spp. (Agro.) mixtures carrying 35S-GFP and the individual constructs indicated in each image. GFP fluorescence was viewed under longwavelength UV light at 7 days postinfiltration (dpi). (G) Northern blot analysis of the steady-state levels of GFP mRNA and siRNA extracted from different infiltrated patches shown in panel A to F. 28 S rRNA and t RNA were used as loading controls for detection of GFP mRNA and GFP siRNA respectively.

As mentioned above, RNA silencing is a multi-step process [17]. To determine in which step Pns6 targets RNA silencing, we tested the effect of Pns6 on GFP dsRNA-triggered silencing. To do this, leaves of transgenic *N. benthamiana *plant line 16c were infiltrated with *Agrobacterium tumefaciens *harboring 35S-ssGFP (sense GFP RNA), 35S-dsGFP (IR-GFP), and a binary vector containing S6, ΔS6, or TAV2b under the control of the 35 S promoter. As shown in Figure 3, leaf patches infiltrated with 35S-ssGFP plus 35S-dsGFP or with 35S-ssGFP plus 35S-dsGFP plus 35S-S6 (or 35S-ΔS6) lost GFP fluorescence at 7 dpi, indicating strong GFP RNA silencing. Consistently, the accumulation of GFP mRNA was hardly detectable in these leaf patches. This suggested that Pns6 could not suppress silencing induced by dsRNA. As expected, TAV2b suppressed GFP RNA silencing triggered by dsRNA, as indicated by the bright green fluorescence and high levels of accumulation of GFP mRNA in leaves infiltrated with 35S-ssGFP plus 35S-dsGFP plus 35S-TAV2b (Figure 3).

**Figure 3:**
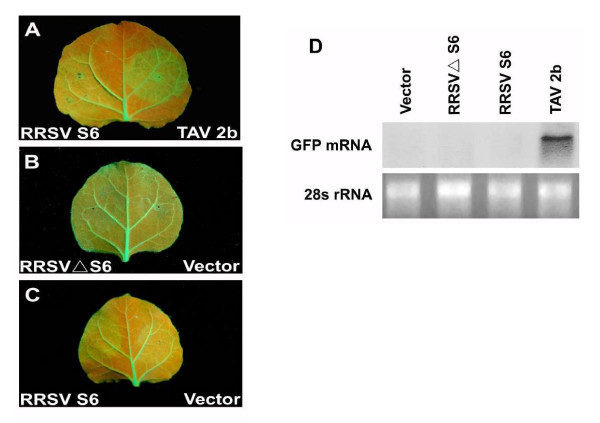
**RRSV Pns6 can not inhibit local silencing induced by dsRNA (A-C) *N***. *benthamiana *line 16c plants were coinfiltrated with *Agrobacterium *spp. (Agro.) mixtures carrying 35S-dsGFP and the individual constructs indicated in each image. GFP fluorescence was viewed under longwavelength UV light at 7 days postinfiltration (dpi). (D) Northern blot analysis of the steady-state levels of GFP mRNA extracted from different infiltrated patches shown in panel A to C. The bottom gel shows rRNA with ethidium bromide staining as a loading control.

These data showed that pns6 of RRSV had silencing suppressor activities. It targeted an initial step of RNA silencing upstream of dsRNA production. This was consistent with a previous report which demonstrated that Pns6 of RRSV had nucleic acid binding activities and preferentially bound ssRNAs [12]. Nucleic acids binding is a common feature of many VSRs. It is possible that Pns6 binds ssRNAs and prevents them from being copied by cellular RDRs. In a primary attempt to exploit this possibility, we generated ΔS6201-273, in which the animo acids from 201 to 273 were deleted (Figure 1). This region of amino acids has been shown to be essential for RNA binding of RRSV pns6 [12]. Indeed, the S6 mutant lost silencing suppressor activity: when co-expressed with GFP in leaves of *N. benthamiana *line 16c, it could not maintain strong GFP fluorescence in infiltrated leaf patches. The GFP mRNA accumulation level was very low at 7dpi., whereas that of GFP siRNAs was markedly high (Figure 2F).

Results from the above experiments indicated that RRSV Pns6 was a silencing suppressor but offered no clues with regards to whether this function would have biological implications for viral infection. The role of Pns6 in RRSV infection can not be tested directly because of the lack of an infectious clone for this virus. Therefore, we utilized pGR107, a PVX vector to express S6 in a heterologous virus. Seedlings of *N. benthamiana *plants (four-to six-leaf stage) were inoculated with PVX, PVX-S6 or PVX-ΔS6, respectively.

Variations in symptom severity were observed in individual infections. However, a general trend was that PVX-S6 elicited more severe symptoms than PVX or PVX-ΔS6 did. This was especially obvious after 9dpi. At this time, only mild chlorotic spots could be observed on some leaves from *N. benthamiana *infected by PVX or PVX-ΔS6. However, most plants infected by PVX-S6 exhibited very severe symptoms, with some newly developed leaves being abnormal in shape (Figure 4A). The symptoms induced by PVX-S6 sustained throughout the life of the plants, whereas only a small proportion of plants infected by PVX or PVX-ΔS6 were symptomatic during later stages of observation. These results showed that RRSV S6 can accentuate symptoms when expressed by a heterologous virus. To correlate this function of RRSV S6 with silencing suppression, the accumulation levels of PVX in systemically infected *N. benthamiana *plants were detected. Northern blot analysis revealed that the concentration of PVX in plants infected by PVX-S6 was very high at 18dpi. In contrast, the presence of PVX RNA was hardly detectable in newly developed leaves from plants infected by PVX or PVX-ΔS6 at this time (Figure 4B). This indicated that RRSV S6 markedly enhanced the replication of PVX, presumably through suppression of RNA silencing. However, it was interesting to note that no differences in the accumulation of PVX RNAs were detected between plants infected by PVX-S6 and those infected by PVX or PVX-ΔS6 at 9dpi (Figure 4B). As plants infected by PVX-S6 showed more severe symptoms than those infected by PVX or PVX-ΔS6 at this time (Figure 4A), this implicated that Pns6 might have direct effects on the normal physiology of the infected plants independent of its ability to enhance viral multiplication.

**Figure 4:**
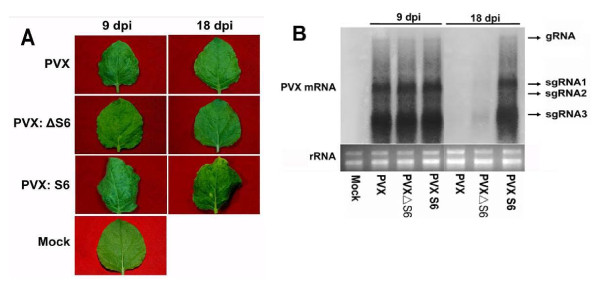
**RRSV Pns6 enhances pathogenicity of chimeric PVX**. (A) plants infected by PVXΔS6 or PVXâ–³S6 show mild disease symptoms as a few scattered chlorotic speckles, whereas leaves infected with PVX-S6 show more severe symptoms. (B) RNA gel blot analysis of accumulation of PVX genomic (gRNA) and subgenomic mRNAs (sgRNA1 to sgRNA3) at 9 and 18 dpi. The bottom gel shows rRNA with ethidium bromide staining as a loading control.

Taken together, we identified pns6, which is composed of 592 amino acids with a molecular weight of about 65 kDa, as a silencing suppressor of RRSV. This was the first VSR to be identified from an oryzavirus. We showed that the RNA binding activities of Pns6 might be important for its silencing suppressor function. Similar studies on other VSRs of plant infecting reoviruses have yet to be done [24-26]. Interestingly, we have recently reported that pns6 might be a cell-to-cell movement protein of RRSV. Pns6 could complement the cell-to-cell movement of the movement-deficient TMV in *N. tabacum Xanthi nc *and *N. benthamiana *plants. When transiently expressed in epidermal cells from *N. benthamiana*, the Pns6-eGFP fusion protein was present predominantly along the cell wall [29]. This was consistent with the notion that virus movement and RNA silencing are intimately related processes. Some well-known VSRs including p19, 2b, Hc-Pro had been implicated in virus long-range movement before the recognition of their silencing suppressor activities [30-33]. For Tobacco Etch Potyvirus HcPro protein, a correlation of silencing suppression and the ability to mediate long-distance virus movement has been demonstrated [34]. Through random mutagenesis of the P25 gene, Bayne et al. showed that suppression of silencing is necessary for cell-to-cell movement of PVX through plasmodesmata [35]. In addition, the movement-deficiency phenotype of a TCV CP deletion mutant could be complemented by a series of silencing suppressors *in Trans *[36]. Thus, our findings may serve as an example in which a protein encoded by a rice virus functions in both RNA silencing and viral movement.

## Competing interests

The authors declare that they have no competing interests.

## Authors' contributions

JW and ZD performed the experiments, analyzed the data, and drafted the manuscript and contributed equally to this work. CW, LC, MH and QL assisted in the performance of the experiments. ZW, YL and LX designed the study, interpreted the data and critically revised the manuscript. All authors read and approved the final manuscript.

## Supplementary Material

Additional file  1 **Materials and Methods**. Word DOC containing the Materials and Methods section. Click here for file

Additional file  2 Table S1: Primers used in this study. Click here for file
