# Supplementary material for: Identification of Pns6, a putative movement protein of RRSV, as a silencing suppressor
Source: Virol J. 2010 Nov 22;7:335. doi: 10.1186/1743-422X-7-335 (PMC3002307; doi:10.1186/1743-422X-7-335)
Supplement: Additional file 1 — Materials and Methods. Word DOC containing the Materials and Methods section. [file 1743-422X-7-335-S1.DOC]

**Materials and methods**

**Plasmids and agrobacterium**. The sequences of all the primers used in this study were listed in Table. 1. The following primer pairs were used to amplify the ORF of RRSV S6, S7, S10 by PCR respectively : S6-F1/ S6-R1, S7-F/S7-R and S10-F/S10-R. The PCR products for each gene were cleaved by *KpnⅠ* and *EcoRⅠ* and then ligated into the vector PZP212, creating 35S-S6, 35S-S7 and 35S-S10, respectively. The plasmids, after verification of their sequence, were transformed into *Agrobacterium tumefaciens.*

S6 ORF was also amplified by using the primer pair S6-F5/S6-R3, which contain cleavage sites for *Smal* and *Sall,* to clone the S6 into pGR107 (an infectious clone of PVX, kindly provided by Dr Baulcombe). The recombinant plasmid was named PVX: S6.

The primer S6-F3 was used to obtain ΔS6. PCR amplification using this primer results in the deletion of the first nucleotide acid of the S6 ORF. The ΔS6 was modulated into the same binary vector as S6, resulting in 35S-ΔS6 . Similarly, the primer S6-F4 was used to create PVX: ΔS6.

To creat S6△201-273AA, overlaping PCR were coducted using the primer pairs S6-R2/S6-F2 and S6-F1/S6-R1.

**Agroinfiltration and GFP imaging** The *N. benthamiana* plants constitutively expressing GFP transgene (line 16c; a gift from David Baulcombe) and the Agrobacterium infiltration operation have been described previously **[7]**. The *N. benthamiana* line 16c plants were cultured in growth chambers at 22 to 24°C before and after infiltration. GFP fluorescence was observed under long-wavelength UV light (Black Ray model B 100A; UV Products) and photographed by using a Nikon D70 digital camera with a Y48 yellow filter.

**RNA extraction and Northern Blot** Total RNAs were extracted from leaves with TRIzol reagent (Invitrogen) according to the manufacturer’s instructions. Northern blotting analysis were conducted according to instructions described in the “The DIG system user’s guide for filter hybridization” For Northern blot analysis of siRNAs, low-molecular-weight RNAs were enriched from total RNAs by eliminating high-molecular-weight RNA using 5% polyethylene glycol (PEG 8000) plus 0.5 M NaCl, separated on a 15% polyacrylamide–7 M urea gel, and transferred to Hybond-N membranes. The hybridization and detection of siRNA were performed as described previously **[24]**. The probes used in the analysis of siRNA were the same as those described above for Northern blots of mRNA.
