# Supplementary material for: Identification of Pns6, a putative movement protein of RRSV, as a silencing suppressor
Source: Virol J. 2010 Nov 22;7:335. doi: 10.1186/1743-422X-7-335 (PMC3002307; doi:10.1186/1743-422X-7-335)
Supplement: Additional file 2 — Table S1: Primers used in this study. [file 1743-422X-7-335-S2.DOC]

**Table 1** Primers used in this study

| **Primer name** | **Primer sepuences** | ***RE*** |
| --- | --- | --- |
| S6-F1 | 5′-ATA *GGT ACC* ATG CAG CTC TTC ATA GTC AAA CTT -3′ | *KpnⅠ* |
| S6-R1 | 5′-CTA *GAA TTC* TCA ATC AAG CTC CTT ACA TTC-3′ | *EcoRⅠ* |
| S7-F | 5′-ATA *GGT ACC* ATG GAC GAG CTA ACT TTA TCC ATT-3′ | *KpnⅠ* |
| S7- R | 5′-ATA *GAA TTC* CTA TCC CTC GAC GGG AGG CCC-3′ | *EcoRⅠ* |
| S10-F | 5′-ATA *GGT ACC* ATG CCT TTC GTG CAA TTC CCG-3′ | *KpnⅠ* |
| S10-R | 5′-ATA *GAA TTC* CTA CTC TGC GTC ATC ACC AAA GTT-3′ | *EcoRⅠ* |
| S6-R2 | 5′-TCT CTA ACC TCT TGG GCT TTA CTT GCC GAA CCA A-3′ |  |
| S6-F2 | 5′-TTG GTT CGG CAA GTA AAG CCC AAG AGG TTA GAG A-3 |  |
| S6-F3 | 5′- GTA *GGT ACC* TGC AGC TCT TCA TAG TCA A -3′ | *KpnⅠ* |
| S6-F4 | 5′- TC*C CCG GG*T GCA GCT CTT CAT AGT CAA ACT -3′ | *SmaⅠ* |
| S6-F5 | 5′- TAC *CCC GGG* ATG CAG CTC TTC ATA GTC AAA CTT -3′ | *SmaⅠ* |
| S6-R3 | 5′- GTA *CGT CGA* CTC AAT CAA GCT CCT TAC ATT CAG GT -3′ | *SalⅠ* |
